# Fusion Transcript Discovery in Formalin-Fixed Paraffin-Embedded Human Breast Cancer Tissues Reveals a Link to Tumor Progression

**DOI:** 10.1371/journal.pone.0094202

**Published:** 2014-04-11

**Authors:** Yan Ma, Ranjana Ambannavar, James Stephans, Jennie Jeong, Andrew Dei Rossi, Mei-Lan Liu, Adam J. Friedman, Jason J. Londry, Richard Abramson, Ellen M. Beasley, Joffre Baker, Samuel Levy, Kunbin Qu

**Affiliations:** Genomic Health Inc., Redwood City, California, United States of America; Baylor College of Medicine, United States of America

## Abstract

The identification of gene fusions promises to play an important role in personalized cancer treatment decisions. Many rare gene fusion events have been identified in fresh frozen solid tumors from common cancers employing next-generation sequencing technology. However the ability to detect transcripts from gene fusions in RNA isolated from formalin-fixed paraffin-embedded (FFPE) tumor tissues, which exist in very large sample repositories for which disease outcome is known, is still limited due to the low complexity of FFPE libraries and the lack of appropriate bioinformatics methods. We sought to develop a bioinformatics method, named gFuse, to detect fusion transcripts in FFPE tumor tissues. An integrated, cohort based strategy has been used in gFuse to examine single-end 50 base pair (bp) reads generated from FFPE RNA-Sequencing (RNA-Seq) datasets employing two breast cancer cohorts of 136 and 76 patients. In total, 118 fusion events were detected transcriptome-wide at base-pair resolution across the 212 samples. We selected 77 candidate fusions based on their biological relevance to cancer and supported 61% of these using TaqMan assays. Direct sequencing of 19 of the fusion sequences identified by TaqMan confirmed them. Three unique fused gene pairs were recurrent across the 212 patients with 6, 3, 2 individuals harboring these fusions respectively. We show here that a high frequency of fusion transcripts detected at the whole transcriptome level correlates with poor outcome (P<0.0005) in human breast cancer patients. This study demonstrates the ability to detect fusion transcripts as biomarkers from archival FFPE tissues, and the potential prognostic value of the fusion transcripts detected.

## Introduction

Oncogenesis is understood to be driven by ten distinctive and interactive capabilities that enable tumor growth and metastasis [1]. One of the underlying hallmarks of cancer cells is genome instability, which fosters random mutations and chromosomal rearrangements. These genomic aberrations, which include translocations, deletions and inversions, can produce oncogenic gene fusions that can be exploited pharmacologically. A classic example of oncogenic fusions is BCR-ABL1 in chronic myelogenous leukemia, which is generated by a translocation between chromosomes 9 and 22 [2], and exhibits constitutive ABL1 tyrosine kinase activity. This discovery led to the development of the targeted tyrosine kinase inhibitor Imatinib approved in 2001 [3]. With advances of modern technology in medicine, the turnover time from discovery of a molecular biomarker to drug approval has been reduced to a period as brief as four years, as demonstrated by the development of Crizotinib treatment for the 2–7% of non-small lung cancer patients possessing the EML4-ALK fusion [4], [5]. Recently, the advent of next-generation sequencing technology has enabled detection of a number of rare recurrent gene fusion events that have potential therapeutic relevance to common solid tumors, including KIF5B-RET, which occurs in about 1% lung adenocarcinomas [6]–[9].

The detection of functional gene fusion events generated by chromosomal translocations has been facilitated by the application of RNA-Seq technologies. Numerous bioinformatics methods are available to detect fusion transcripts from RNA-Seq paired-end read data (ChimeranScan [10], SnowShoes-FTD [11], GSTRUCT-fusions [12] and GFP [9]) or single-end read (TopHat-Fusion [13], FusionMap [14] and FusionFinder [15]). All fusion transcript detection methods utilize split reads, in which a single-end read or one read from the pair-end read is mapped to each end of two fused genes exactly at the fusion junction site. In addition to split reads, paired-end approaches take advantage of bridging reads in which each read is mapped to each of the fused genes independently, thus providing extra evidence for the existence of a fusion junction than split reads alone. Most of these published methods evaluate RNA prepared from cell lines or fresh frozen tumor tissue from biopsy or resection. RNA from these sources is generally relatively intact and produces longer insert size libraries for sequencing, which greatly facilitates the detection of fusion transcripts.

The standard clinical practice of creating FFPE tissue specimens from biopsies and surgical resections has generated very large numbers of FFPE tissue blocks in pathology archives that have associated, metadata-rich, long term clinical records. Therefore, the detection of fusion transcripts in FFPE tissues may reveal fusion transcripts of clinical relevance. Any attempts to detect fusion transcripts from FFPE tissues must address the extensive RNA fragmentation that occurs during storage of FFPE blocks and continues as block archival age increases [16], and also the substantial amounts of precursor RNAs detected in this tissue source [17]. As a result, FFPE RNA-Seq libraries have short insert sizes, low complexity (i.e., many short sequence segments with identical nucleotide composition) and a large amount of intronic sequence [17]. Difficulties accurately trimming the sequencing adaptor at the 3′-end of reads from FFPE samples as well as the chemical modifications of RNA during formalin treatment can also decrease mapping quality such that the mapping rates from FFPE RNA-Seq libraries are lower than those from fresh frozen tissues. As a result of RNA fragmentation in FFPE tissue, whereby a median RNA fragment size of 100 bp is found, we reasoned that 50 bp single-end reads would provide a robust cost-effective sampling methodology for our study. We describe here the development and application of a bioinformatics method, gFuse, for the detection of fusion transcripts in RNA-Seq data from archival FFPE samples. This method addresses the challenges outlined and employs short sequence single-end reads (50 bp) enabling a cost effective method of analyzing large numbers of FFPE samples.

In addition to sequence information, expression profiles have been used to provide supporting evidence for fusion transcripts. The utilization of expression data for fusion transcript detection is a feature of the COPA (Cancer Outlier Profiling Analysis) method that was devised for analysis of microarray databases [18]. Cancer-related genes identified as expression outliers in microarray experiments led to the discovery of TMPRSS2 fused to ETS transcription factors, the first known recurrent gene fusions in common solid carcinomas. Fusion RNAs are expected to exhibit a marked expression discontinuity between the preserved side and discarded side of a given fusion junction, compared to expression of these genes in samples without the fusion transcript. Recently published fusions detected using RNA-Seq data have displayed this discrete expression pattern at acceptor fusion junction sites [8], [9]. Multiple bioinformatics approaches including FusionSeq [19], deFuse [20] and TopHat-Fusion [13] have used expression data in their pipelines and all these methods rely on the analysis of an individual subject. The cohort-based approach described here compares expression levels across a cohort of subjects, combined with exon/intron level expression interruption, to identify putative fusion transcripts. Due to the large proportion of sequences (65% of uniquely mapped reads) that map to introns in FFPE RNA-Seq data [17], we included reads mapped to the introns to comprehensively measure expression of each gene.

In this study, we detected fusion transcripts in two breast cancer cohorts, the Providence cohort of 136 patients and the Rush cohort of 76 patients with average FFPE block archive ages of 8.5 years and 13.4 years respectively [17], [21]. These two cohorts have been previously used in the development of a 21-gene qRT-PCR breast cancer recurrence risk assay [21], [22]. Recently, the whole transcriptome RNA-Seq analysis of the Providence cohort has demonstrated that the technology used is sensitive and specific [17]. Here, we apply these single-end 50 bp RNA-Seq data to identify fusion transcripts and relate them to breast cancer prognosis.

## Materials and Methods

### Breast cancer patients and RNA-Seq dataset

One hundred thirty-six primary breast cancer FFPE tumor specimens with clinical outcomes were provided by Providence St. Joseph Medical Center (Burbank, CA), with institutional review board approval [22]. The clinical characteristics, RNA-Seq sample preparation and sequencing of the Providence cohort of 136 primary breast cancer FFPE tumor specimens were described earlier [17]. Briefly, total RNA was isolated from three 10-µm FFPE tissue sections per patient using Epicentre's MasterPure Purification Kit (Epicenter Biotechnologies, Madison, WI). Paraffin was first removed by xylene extraction followed by ethanol wash. A DNase I treatment step was included to remove DNA from total nucleic acids. The same procedure was employed for RNA isolation from a second breast cancer study cohort from Rush University Medical Center. Seventy-eight primary breast cancer FFPE tumor specimens with clinical outcomes were provided by Rush University Medical Center (Chicago, IL), with institutional review board approval [21]. The same method of sample preparation [21] and sequencing [17] was applied to 76 of 78 Rush samples. Two remaining Rush samples did not yield enough RNA for sequencing. Directional RNA-Seq libraries were prepared using ScriptSeq RNA-Seq Library Preparation Kit (Epicenter Biotechnologies, Madison, WI) as described previously [17]. The quality of the RNA-Seq libraries was assessed using Agilent DNA Kits on a 2100 Bioanalyzer instrument (Santa Clara, CA). Sequence reads of 50 bp in length were processed by CASAVA, the standard Illumina package, and data quality assessment was described earlier [17]. The definition of clinical recurrence in these patients was determined as in the original study plans [21].

### Fusion transcript detection pipeline gFuse

We define a fusion junction as a unique pair of donor and acceptor genomic positions such as “+chr17:5250220->+chr17:11532734”, and a fusion or fusion event as an occurrence of a particular fusion junction within a patient sample. The definition of symbols used to define each junction is: “->” indicates the splicing direction from donor to acceptor, “+” indicates the transcription direction on the top of chromosome strand, and “-” indicates the transcription direction on the bottom of the chromosome strand. The donor genomic position is the last base of the preserved side of the donor and the acceptor genomic position is the first base of the preserved side of the acceptor.

**Figure 1:**
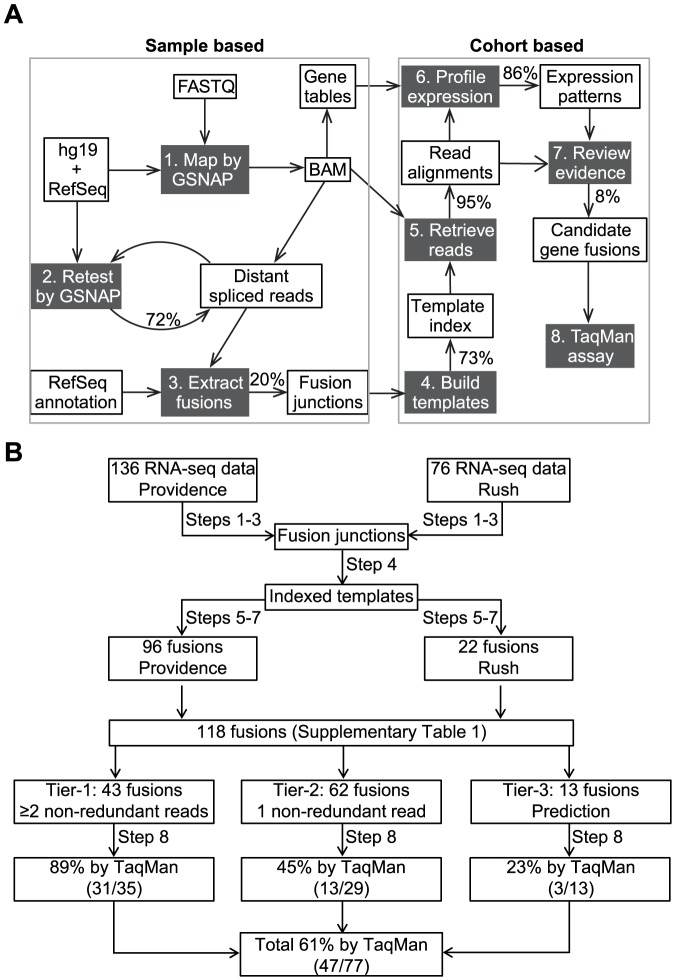
The schema and workflow of our fusion detection pipeline gFuse, illustrated for two breast cancer cohorts. A. The sample and cohort based strategies are integrated in RNA-Seq fusion transcript detection. Each step of the pipeline is numbered in shade, and explained in Materials and Methods. The percentages show the fusion junctions retained after each step in all Providence samples. B. Dataflow and main results of fusion events detected in Providence and Rush are shown side-by-side with each step corresponding to the numbered step in Figure 1A. The numbers of fusion events selected for TaqMan assays and the TaqMan supported ones are in parentheses.

The fusion transcript detection pipeline gFuse consists of two strategies, a sample-based strategy and a cohort-based strategy (Figure 1A). The sample-based strategy interrogates each RNA-Seq sample individually and nominates candidate fusion junctions. The cohort-based strategy has two features that take advantage of the cohort-based information. The first feature is to combine the candidate fusion junctions in the beginning step of the cohort based analysis, which increases the chance of identifying recurrent fusion transcripts across the two cohorts studied here. The second feature is to confirm the presence of each fusion candidate in each individual sample across the whole cohort by examining read alignment and expression profiling evidence. The pipeline was developed in Linux Shell, Perl and R languages, and the data processing was on a Linux cluster. The detailed steps of gFuse (Figure 1A) are described below.

#### Step 1: Map by GSNAP

Raw FASTQ sequencing data from the Providence and Rush cohorts were generated using CASAVA software. The FASTQ files were mapped to the human genome (version GHCh37/hg19) along with RefSeq splicing sites and dbSNP database (version 135) using the RNA-Seq aligner GSNAP [23]. An important feature of GSNAP is its ability to detect a distant splice junction within a single read. Local splice junctions derive from splicing events within a single gene in a consistent transcription direction, whereas distant spliced junctions derive from splicing events between different genes or chromosomes. Distant splicing events can also include splicing events occurring within the same gene, but in the opposite transcription direction [24].

Two filters were installed to remove low quality and unwanted reads. The quality filter retained reads with a minimum 15 bases at any position with a base quality score of 20 or above. To filter out the un-wanted reads, a number of abundant sequences including biological sequences (e.g., ribosomal RNA and mitochondrial sequences), and sequences introduced during library prep (e.g., PhiX) were removed from alignment (BAM) files. Only reads passing both filtering thresholds and uniquely mapped to human genome were retained to calculate the gene feature counts that provide expression values for exonic and intronic regions. The gene feature count is the number of aligned bases from reads mapped within the feature region. These gene feature counts are referred to as “gene tables” in Figure 1A.

#### Step 2: Retest by GSNAP

In order to remove false positives, potential distant spliced reads in Step 1 were re-tested using GSNAP parameters that favor local alignment. Each alignment from the GSNAP re-run was examined, any reads meeting all of the following criteria were considered false positive distant splicing reads in the original GSNAP output, and removed from further analyses: the total matched length in the local alignment was at least 44 bp with a gap alignment tolerance of 1 bp. Reads that successfully passed through this step were considered to include a distant spliced junction.

#### Step 3: Extract Fusions

The resulting distant splicing junctions were then annotated and candidate fusion transcripts were selected. Specifically, the alignments of reads that passed Step 2 were examined, and reads with any mismatches within 5 bp of the distant splicing junction site or mapped to the anti-sense strand of annotated genes were removed. Anti-sense reads were removed in this step since directional RNA-Seq libraries were constructed in the two cohorts analyzed here. The remaining reads were grouped according to the distant splicing junction sites, and each junction site was annotated based on the University of California, Santa Cruz RefSeq sequence annotation (ftp://hgdownload.cse.ucsc.edu/goldenPath/hg19/database/refGene.txt.gz). During this annotation step, any junctions mapped to pseudo-genes, un-annotated gene regions, or multiply mapped RefSeq genes were removed. Also, gene rearrangements within the same gene or potential transcript read-throughs were also eliminated. At this stage, candidate fusions met at least one of the following criteria: (1) they mapped to different chromosomes, (2) they mapped to different RefSeq genes, (3) they were in opposite directions on same chromosome, or (4) they were at least 1 MB apart if on the same chromosome.

#### Step 4: Build Templates

**Figure 2:**
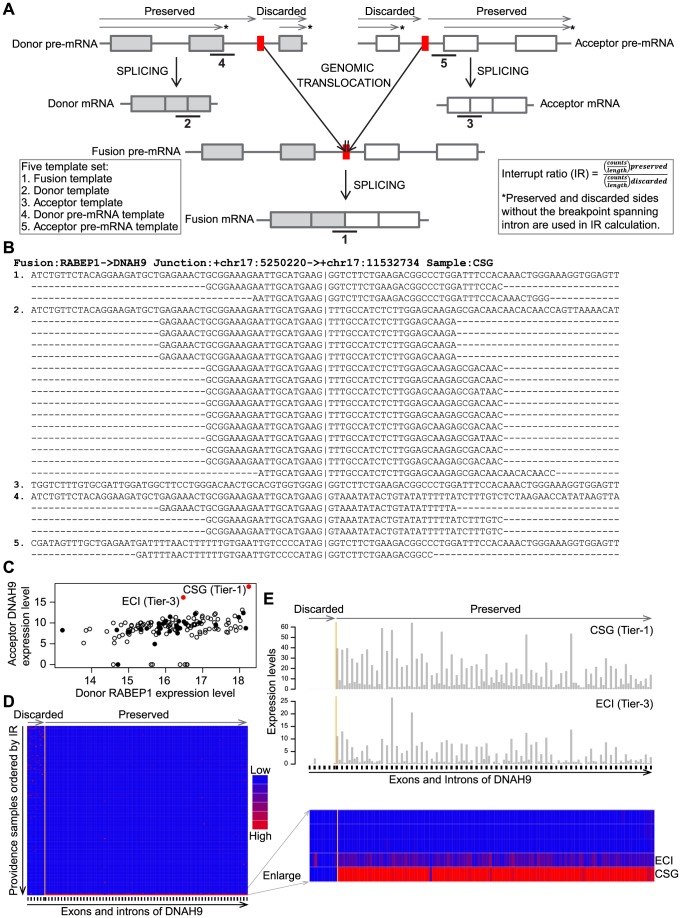
The utilization of a five template set and expression profiling for fusion transcript detection. A. The concept of five template set is illustrated with six RNA transcripts for a fusion transcript in a FFPE RNA sample. Each template is numbered under lines around the corresponding RNA sequence. The preserved and discarded sides of donor or acceptor are indicated by arrowed lines indicating transcription directions above each pre-mRNA. The red blocks are DNA breakpoints. The interrupt ratio (IR) is calculated by using the * marked preserved and discarded sides accordingly for donor or acceptor fusion genes. B. All supporting RNA-Seq split reads are aligned to five templates of fusion RABEP1->DNAH9 in the Providence sample CSG. Each template is numbered according to Figure 1A. The vertical line indicates the junction site. C. Two samples are shown as outliers (solid red dots) when the gene expression levels of donor RABEP1 are plotted against acceptor DNAH9 in the Providence cohort. The expression levels are log2 base counts normalized by library size factors. TaqMan tested negative samples are labeled as solid black dots. D. Exon and intron expression levels of acceptor DNAH9 in the Providence cohort show the interrupted expression pattern in samples CSG and ECI at the predicted fusion junction site (orange line). The base counts of each exon and intron are normalized by library size, then center-scaled across the Providence cohort. The vertical arrow indicates RNA samples from low to high IR values of DNAH9. The exons (black ticks) and introns of DNAH9 are ordered according to the transcription direction (horizontal arrow), with the intron harboring DNA breakpoint omitted in Figure 2D and 2E. E. The base counts of exons and introns of acceptor DNAH9 in two samples show interrupted expression patterns at the fusion junction site. The base counts are normalized by library size then divided by length of each exon or intron.

At this stage, fusion junctions from both the Providence and Rush cohorts were combined (Figure 1B). In order to remove false positives introduced by homologous sequences around candidate fusion junctions and to enable accurate mapping of supporting reads, a five template set was created for each candidate fusion. The features of the five template set are depicted in Figure 2A. The set included the following individual templates, each of which included 100 bp of sequence. The set templates were 100 bp, with 50 bp on either side of the candidate junction for fusion templates, because our read length is 50 bp.

- Fusion template: The 50 bp exonic sequence of the preserved region of donor gene plus 50 bp exonic sequence of the preserved region of acceptor gene,
- Donor template: The 50 bp exonic sequence of the preserved region of donor gene plus 50 bp exonic sequence of the discarded region of donor gene,
- Acceptor template: The 50 bp exonic sequence of the discarded region of acceptor gene plus 50 bp exonic sequence of the preserved region of acceptor gene,
- Donor pre-mRNA template: The 50 bp upstream genomic sequence of donor splicing site plus 50 bp downstream genomic sequence of donor splicing site,
- Acceptor pre-mRNA template: The 50 bp upstream genomic sequence of acceptor splicing site plus 50 bp downstream genomic sequence of acceptor splicing site.

Donor and acceptor mRNA or pre-mRNA containing template sequences were used as controls. Since the DNA breakpoints were unknown in RNA-Seq data, a fusion pre-mRNA template could not be created. The genomic sequences were used to generate the pre-mRNA template sequences, and RefSeq sequences were used to generate mRNA template sequences. The sequence of each template in the five template set was retrieved and annotated for each candidate fusion transcript. Candidate fusion junctions were removed if any of their 100 bp templates had the identical sequence with any other template set. BLAST (http://blast.ncbi.nlm.nih.gov/Blast.cgi, version 2.2.25) was used to investigate the homology of the remaining candidate fusions. A separate collection of 300 bp template set was built for each of the fusion junction candidates with the same strategy as described above to provide sequence input to probe designs for qRT-PCR experiments. Homologies between the 300 bp donor template and the 300 bp acceptor template, as well as homologies between the 300 bp donor pre-mRNA template and the 300 bp acceptor pre-mRNA template were evaluated. Any candidate fusion satisfying the following criteria was removed from further analysis: (1) sequence identity of more than 14 bp (empirically determined to effectively remove homologous genes) of 300 bp of the donor template and acceptor template; (2) sequence identity of more than 14 bp of 300 bp of the donor genomic template and acceptor genomic template; and (3) less than 50 bp exonic sequence on either side of fusion, donor, or acceptor template sequences.

The 100 bp five template sets for each of the remaining candidate fusions were used to create a template index using a tool from the GSNAP package.

#### Step 5: Retrieve Reads

In order to increase the sensitivity and to determine the final supporting reads for each candidate junctions, all reads mapped near any junction site based on the genomic location of all candidate fusion template sets and reads not mapped in the original GSNAP BAM file for each RNA-Seq library were selected. The selected reads were re-mapped into the five template set index with GSNAP with the splicing detection parameter turned off. Only good quality reads uniquely mapped to the fusion template were kept.

#### Step 6: Profile Expression

In order to assess the expression of each fusion transcript with a cohort, both exons and introns present in candidate fusions that had at least one read across the fusion junction site were assessed for each donor and acceptor. The gene table including exons and introns derived from Step 1 was normalized by library size factors as described by R package DEseq [25]. The intron immediately before the splicing site on the acceptor gene or the intron immediately after the splicing site on the donor gene were excluded from expression analyses due to uncertainty of the breaking point in the intron (Figure 2A). Exons or introns having counts below 5 reads were padded to 5 reads. The expression Interrupt Ratios (IR) of normalized counts between preserved and discarded sides were calculated for donor and acceptor genes for each sample according to the following formula:
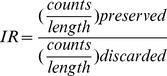



The normalized expression counts of exons and introns in each fusion transcript across samples in a cohort were ordered according to IR values, and a heatmap representing the gene features of the predicted fusion transcript within the cohort was generated. Expression profiling results for an example candidate fusions are shown in Figure 2D of the Results section.

#### Step 7: Review Evidence

Fusions were classified into these three tiers based on evidence (Figure 1B). Data were manually reviewed to classify candidate fusions. The following rules were then applied to rank candidate fusions into three tiers:

- Fusions with a minimum of two non-redundant reads spanning fusion junctions and were kept as Tier-1 fusions regardless of the expression profiling (Figure 1B);
- For fusions with only one non-redundant read, expression profiling evidence was reviewed to select candidates with favorable expression evidence, and ranked as Tier-2;
- Fusions were classified as Tier-3 if they were predicted without any read evidence, but sharing a similar expression profiling with a TaqMan supported fusion after Step 8 described below;
- Multiple samples sharing the same fusion junctions, but without good expression evidence were removed.

A total of 100 unique fusion junctions with 118 fusion events were identified, the full list is available in Table S1.

#### Step 8: TaqMan assay

Quantitative RT-PCR analysis using TaqMan RT-PCR was used to investigate the selected 60 fusion junctions. Reverse transcription was carried out using the Omniscript RT Kit (Qiagen) by incubating amplified RNA with random hexamers and gene-specific primers at 37°C for 1 hour. Primer, probe, and amplicon sequences are shown in Table S2. Fluorogenic probes were dual-labeled with 5′-FAM as a reporter and 3′-BHQ-2 as a quencher. Primers and probes were designed using the Primer3 program restricting amplicon sizes to 65-85 bps (http://frodo.wi.mit.edu/). When Primer3 failed, primer and probe sequences were optimized manually to ensure optimal performance of the TaqMan assay design for the chimeric transcripts. Reverse transcription reaction in the absence of RNA template (i.e., water) was always used as a negative control in all assays. The samples that were previously identified as positive or negative for a particular fusion junction served as controls when needed. Since the RT reaction was multiplexed by using a pooled gene specific primer set, the cDNA derived from a RNA sample was tested with all fusion gene qPCR assays within an assayed gene set. All RNA samples were assayed in triplicate qPCR reactions with 10 µl per well. Thermal cycling conditions were standard for all assays (A heat activation step of 95°C for 10 minutes followed by 40 cycles of 95°C for 20 seconds and 60°C for 45 seconds). All TaqMan assay results including primer and probe sequences are listed in Table S2.

### Fusion confirmation by Personal Genome Machine (PGM, Life Technologies)

Nineteen qPCR supported fusion transcripts were selected to be sequenced on the semi-conductor based Ion Torrent Personal Genome machine (PGM) to confirm the results from qPCR. The selection priority was given to those either recurred in multiple patients or appeared within a single patient as one of the multiple fusion transcripts.

Eight replicate wells of PCR products were generated for each of the fusion targets (19 in total) in 12 Providence/RUSH amplified RNA samples in order to prepare enough PCR product for PGM sequencing of the selected gene fusion candidates. Quantitative RT-PCR analysis using TaqMan RT PCR was used to confirm the presence of PCR product before proceeding to PGM sequencing. Reverse transcription was carried out as described in Step 8. The eight replicate wells of PCR product were pooled for each fusion target. Each PCR product was then purified using 1.8× volume of Agencourt AMPure XP beads (Beckman Coulter), and quality checked and quantitated using the Agilent High Sensitivity DNA Kit (Agilent Technologies). Fusion PCR products from the same patient samples were then pooled together. The Fusion PCR products were then prepared for sequencing using the Ion Plus Fragment Library Kit (Life Technologies) and barcoded using the Ion Xpress Barcode Adapters 1–16 (Life Technologies). The library was amplified with 7 cycles after adapter ligation and cleanup, as required by the protocol. The libraries were individually quantitated using the Agilent High Sensitivity DNA Kit (Agilent Technologies) and diluted to a target concentration of 26 pM. The libraries were pooled in equi-molar quantities prior to emulsion PCR on the Ion OneTouch 2 System (Life Technologies) and subsequently sequenced on a PGM 314 Chip Kit v2 (Life Technologies) using 260 flows.

Ion Torrent Suite software was used to generate FASTQ files in which the barcode adaptors and 3′ end low quality sequences were removed as recommended. To recover read sequences longer than the desired 100 bp in a case of an expected amplicon of 126 bp, the 3′ end quality trimming was turned off for this design. All reads were mapped to the 5 template set sequence database containing the fusion templates. For each of expected fusion amplicons in a given sample, the most abundant reads mapped to the fusion template was selected as the PCR amplicon. The sequence of this read was compared to the sequence of the expected amplicon. If the PCR amplicon matches the expected fusion amplicon, the fusion junction sequence is considered as confirmed.

### Survival analysis

Patients were stratified into different categories based on the fusion number detected. The time to disease recurrence as defined in the original studies [21], [22] was used to generate the Kaplan-Meier plot using the R package Survival.

### Data access

The read alignments which support the fusion transcripts for Providence and Rush cohorts are deposited into Dryad Digital Repository (http://doi.org/10.5061/dryad.98m0m).

## Results

### Fusion transcripts were detected by gFuse, an integrated cohort-based approach

Overall, 118 fusion events, representing 100 unique fusion junctions, were identified in the two cohorts (Table S1). Forty three of the fusion junctions are predicted to produce in-frame chimeric proteins. Based on gene associations with cancer, we selected a total of 60 fusion junction candidates, and designed qRT-PCR assays for these fusion transcripts. Some of the candidate fusions selected for TaqMan assay were observed in 2 or more samples. Therefore by using 60 designs, we tested 77 candidate fusion events by quantitative RT-PCR in amplified RNA from selected patients harboring the corresponding candidate fusions (Table S2). A total of 47 of the 77 fusion events (61%) were supported by TaqMan across the two cohorts irrespective of the sequence evidence. The Tier-1 category of candidate fusions (see Materials and Methods for definitions of Tiers), which have the strongest sequence evidence have the highest support frequency rate (89%). Tier-2 candidates, selected based on the combination of sequence (single read coverage only) and expression profiling, have a 45% support frequency rate. Tier-3 candidates, purely predicted from gene expression patterns, have the lowest support frequency at 23% (Figures 1B). Thus, the TaqMan results are consistent with the level of evidence observed for the three different tiers of fusion candidates. To further confirm fusion junction identified by TaqMan assays, a total of 19 fusion events identified by TaqMan were selected for PGM sequencing. Fusion junctions were amplified by using TaqMan primers, and PCR products containing fusion amplicons were sequenced on the PGM. In all 19 PCR reactions, the PCR amplicons matched the predicted fusion junction sequences (Table 1 and Table S3). In 7 PGM libraries in which a single barcode was used for a single PCR reaction, the amplicon reads represent the most prevalent clonal population in each library indicating that the PCR reactions are specific for these fusion junctions (Table 1).

**Table 1:** Fusion junctions are confirmed by PGM sequencing of PCR amplicons.

Sample	Fusion	Junction	Tier	Number of amplicon reads
HM1	ESR1->AKAP12	+chr6:152265643->+chr6:151669846	Tier-1	252
HM1	ESR1->C6orf211	+chr6:152129499->+chr6:151785588	Tier-2	9702
MJG	TFG->GPR128	+chr3:100438902->+chr3:100348442	Tier-1	5009
MJG	SEMA4C->BRE	-chr2:97527316->+chr2:28561317	Tier-1	4853
ECI	TFG->GPR128	+chr3:100438902->+chr3:100348442	Tier-3	2297
ECI	RABEP1->DNAH9	+chr17:5250220->+chr17:11532734	Tier-3	1927
ECI	ERBB2->IKZF3	+chr17:37868701->-chr17:37949186	Tier-1	2218
CSG	RABEP1->DNAH9	+chr17:5250220->+chr17:11532734	Tier-1	7161#
D87	TFG->GPR128	+chr3:100438902->+chr3:100348442	Tier-2	6187#
II6	ESR1->AKAP12	+chr6:152201906->+chr6:151669846	Tier-2	6880#
IYM	ESR1->AKAP12	+chr6:152201906->+chr6:151669846	Tier-1	9916#
JGV	TFG->GPR128	+chr3:100438902->+chr3:100348442	Tier-3	6108#
L43	TFG->GPR128	+chr3:100438902->+chr3:100348442	Tier-2	5979#
MGM	TFG->GPR128	+chr3:100438902->+chr3:100348442	Tier-2	7499#
DAP	RIMS2->DPYS	+chr8:104709524->-chr8:105436617	Tier-1	5440
DAP	PREX1->SLC9A8	-chr20:47324798->+chr20:48431545	Tier-1	5809
GQW	TANC2->RDM1	+chr17:61086987->-chr17:34247276	Tier-1	1000
GQW	DDX5->IQCG	-chr17:62496667->-chr3:197640913	Tier-1	1919
GQW	EIF4A3->TSPEAR	-chr17:78120592->-chr21:45953806	Tier-2	1296

# In these 7 PGM libraries containing a single PCR reaction with an unique PGM barcode, the fusion amplicons identify the most prevalent clonal population in the library. The detailed experimental results including amplicon sequences are in Table S3.

The underlying fusion transcript method is based on the detection of distant splicing within a single read feature as detected by the RNA-Seq aligner GSNAP [24]. The utility of GSNAP for fusion transcript detection has been demonstrated in fusion transcript detection methods such as GSTRUCT-fusions and GFP [9], [12]. Both of these methods depend on GSNAP to provide fusion read candidates, and then apply a set of filtering modules to remove false positives in paired-end RNA-Seq datasets. To compensate for the short FFPE RNA length, we leveraged data from the two patient cohorts as shown in Figure 1A. The sample-based strategy interrogates each RNA-Seq sample individually and nominates candidate fusion junctions for the following cohort-based analysis, which confirms the presence of each fusion candidate in each individual sample across the whole cohort by examining read alignment and expression profiling evidence. To increase the chance of identifying recurrent fusion transcripts across the cohorts, fusion candidate templates provided by the sample-based strategy were combined in the beginning step of the cohort based analysis. However, in recognition of inter-cohort differences in block archive ages and library quality, the expression profiling step was carried out separately within each cohort (Figure 1B). The average insert size and complexity of the Providence cohort libraries are higher than those of the Rush cohort libraries. Here we describe results from the Providence RNA-Seq dataset [17] to illustrate the performance of the cohort-based computational approach.

Briefly, 50 bp single end reads were mapped to the human reference genome to provide candidate reads splitting across potential fusion junctions similar to GSTRUCT-fusion and GFP (Figure 1A). The candidate fusion split reads were re-mapped against the human reference genome under the GSNAP parameters favoring local alignments. Any reads that aligned locally, and were therefore not split across the fusion junction, were discarded. This alignment re-testing step eliminated 28% of distant spliced junctions identified in Step 1. The RefSeq annotation file was used to annotate these distant spliced junctions. Only junctions mapping to two different annotated genes were kept, and 80% of distant spliced junctions identified in Step 2 were eliminated during the annotation step.

Next, candidate fusion junctions having at least one supporting read were combined from the two cohorts and further tested using the cohort based strategy. The donor and acceptor mRNA or pre-mRNA template sequences were used as controls for the sequence homology search and to generate read alignments in the cohort based approach. This step removed 27% of potential false positive fusion junctions from Step 3. The remaining five template sets were combined and constructed into a single template index. All short reads mapping near any junction sites in the template index as well as reads not mapped in Step 1 were aligned to the template index for each RNA-Seq library. Fusion templates with at least one supporting short read were selected for further cohort based analysis. There are 3 tiers of candidate fusion transcripts generated by gFuse, Tier-1, Tier-2 and Tier-3. The supporting evidence for Tier-1 transcripts is strong while Tier-3 transcripts have weak evidence. Any fusions with at least 2 non-redundant reads across the fusion junctions are defined as Tier-1. Both Tier-2 and Tier-3 were selected based on the expression profiling described below; Tier-2 consists of fusions with a single non-redundant read across the fusion junction and Tier-3 represents predicted recurrent fusions with no read across the putative fusion junction.

The expression profiling step can nominate candidate fusions despite the existence of very limited reads. In fact, here we used the expression profile data to predict known fusions in samples having no detected fusion sequences as illustrated by the example fusion RABEP1->DNAH9 (Figure 2). This fusion junction was initially found in a single Providence sample (CSG) as a Tier-1 fusion with 2 split reads (Figure 2B). In this Tier-1 fusion, there are a total 17 reads across the donor RABEP1 mRNA and pre-mRNA template junctions, and 1 read across the acceptor DNAH9 mRNA and pre-mRNA template junctions. This evidence suggests that the strong donor promoter drives the expression of fusion transcripts. Consistent with the read coverage around junction sites, this fusion also appears as one of two expression outliers in the Providence cohort (Figure 2C). A second patient (ECI) is the only other patient that appears to have the same discrete expression pattern which exists in the sample CSG as evidenced by examination of the exon/intron expression levels of acceptor DNAH9 across the Providence cohort (Figure 2D). The samples in the cohort were ordered by IR (defined in the Materials and Methods section) to facilitate the expression outlier identification. The individual exon/intron expression levels of DNAH9 also show the discrete expression patterns around fusion junction site in two Providence samples (Figure 2E). Therefore, we assigned the sample ECI as a Tier-3 candidate for fusion of RABEP1->DNAH9, even in the absence of reads across fusion junction in ECI. Both fusion events were supported by TaqMan with an average C_T_ of 30.11 (CSG) and 34.86 (ECI) respectively, while other 39 samples tested were negative in the assay (Figure 2C). Therefore we conclude that there are two fusions or recurrent fusion events associated with a particular fusion junction “+chr17:5250220 -> +chr17:11532734” in the Providence cohort (Figures 2C, D and E).

### Fusion partners are cancer related genes

The majority of fusion junctions are intra-chromosomal genomic rearrangements (69 out of total 100 fusion junctions), consistent with findings of others [10], [26]. Of the 100 unique fusion junctions, only TFG->GPR128 had been described previously [11], [27], [28]. It is noteworthy that a few of these fusion junctions are detected in both of the examined patient cohorts. Three recurrent fusion transcripts including TFG->GPR128, ESR1->AKAP12 and RABEP1->DNAH9 were supported by TaqMan assays using amplified RNA from 6, 3 and 2 patients respectively, in the two cohorts of 212 total patients. Interestingly, among three ESR1->AKAP12 fusion events in three different patients, there are two unique fusion junctions sharing the same acceptor junction site but differing at the donor junction sites by one exon. Since both these ESR1->AKAP12 fusion junctions are in frame and the differing ESR1 exon doesn't harbor any known functional domains (Figure S1A), these two fusion transcripts may possess the same biological function. Both fusion protein isoforms replace the ESR1 ligand binding site with functional domains from AKAP12 (Figures S1B and S1C). The lost ligand binding site of ESR1 is known to interact with another AKAP family member AKAP13 [29]. AKAP12 is a scaffold protein in signal transduction with tumor suppressor activities, and present in the plasma membrane, cytosol or endoplasmic reticulum [30]. The function of AKAP12 to organize the protein kinase A and C at these biological relevant locations might be disrupted if its location is changed. The fused AKAP12 protein might have different cellular localization and thus possess modified functions from the wild type AKAP12. In addition, both fusion protein isoforms may cause constitutive ligand-independent signaling. As a result, the patients harboring ESR1->AKAP12 fusion may exhibit different responses to breast cancer hormone therapy.

On the other hand, in certain fusion cases we identified varied junctions between two identical fused partners within a single patient. One patient in the Providence cohort has three different ERBB2->IKZF3 junctions that only differ at the donor junction site, and one patient in the Rush cohort has two different TRIM37->BCAS3 junctions that only differ at the donor junction site (Table S1). In these two cases qRT-PCR assays were designed to the junction sequences with the greatest number of RNA-Seq reads, and the dominant fusion junctions were supported by TaqMan in each case. Also, multiple recurrent partners fused to different gene partners were identified in our dataset, and supported by TaqMan assay: one tumor harboring ESR1->AKAP12, another with the fusion gene ESR1->C6orf211; LRP5 fused to different acceptors KAT6A and SLC22A24 in the same tumor; ADK as an acceptor in the fusion DLG5->ADK in one patient, and as a donor in the fusion ADK->C10orf11 in another patient; similarly, ACACA as the donor of ACACA->MSI2 in one patient, and as the acceptor of UTP18->ACACA in another patient.

We searched the Mitelman fusion database with all 184 unique fusion partners including donors and acceptors from the final 118 fusion list, and 29 partners were found fused to various different partners in that database [28]. The statistically significant enrichment of Mitelman fusion genes (Fisher's test P = 7×e^−8^) is 3.5 fold compared to all known RefSeq genes. Among them, ACACA, BCAS3, DDX5, FBXL20, IKZF3, RAF1, TFG and TRPS1 were fused to more than one partner in the database. These observations suggest fusion events are unlikely to be random although they appear to be rare in solid tumors.

The identified fusion partners tend to be cancer-related: 82% of the total 83 fusion junctions (96 fusion events in Figure 1B) identified from the Providence cohort have at least one partner in COSMIC database, which contains sequences of many genes frequently altered in cancers. This is consistent with other evidence for frequently mutated genes prone to genomic rearrangements in the cancer genomes [9]. The discovery of fusion transcripts containing partners that regulate repair of DNA double-strand breaks and homologous recombination, such as RAD21, RDM1, BRCA2 and SHFM1, is consistent with abundant evidence for aberrant regulation of DNA replication in cancer.

### Higher numbers of fusion events are associated with poor tumor prognosis

The average number of fusion events detected per patient across Providence and Rush cohorts is 0.63 and 0.29, respectively, far less than the average of 4.2 fusions reported in fresh frozen breast cancer biopsies [10], [11]. This difference can reasonably be attributed to the poor quality of FFPE RNA, and a resulting limit on our ability to comprehensively detect fusion events in these samples. Between the Providence and Rush data sets, the latter has older archival ages, poorer quality RNA, and yields far fewer identified fusion transcripts (Figure 1B).

**Figure 3:**
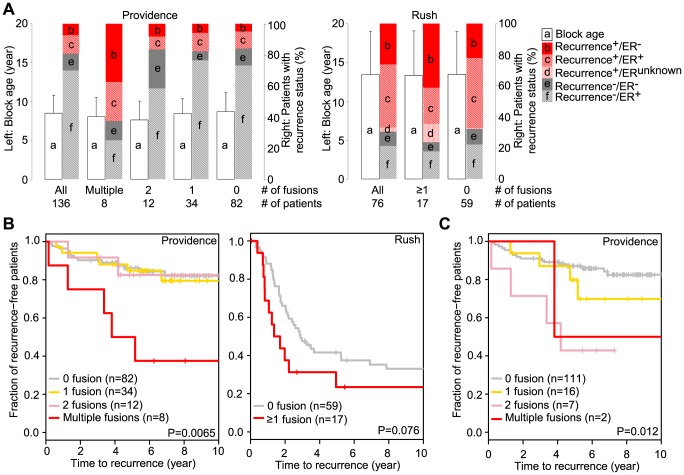
Breast cancers with high fusion frequency have poor prognosis. A. The distributions of block age, clinical recurrence and ER status are shown according to fusion number categories in Providence and Rush cohorts. The archived block age is plotted as mean and standard deviation for each category. ER status was assessed by immunohistochemistry. The patient number for each category is labeled accordingly. B. Kaplan-Meier plots of each fusion number category show Providence patients with multiple fusions had poor prognosis, and a similar trend exists for Rush patients. The log-rank p-values are indicated in Kaplan-Meier plots. C. Kaplan-Meier plot with the 36 TaqMan supported fusion transcripts in the Providence cohort. There are another 11 TaqMan supported fusion transcripts from the Rush cohort but they are too few to generate a meaningful Kaplan-Meier plot.

Within each patient cohort we stratified patients according to the number of fusion events (Figure 3A) to determine whether the number of fusion events detected within individual tumors related to the likelihood of disease recurrence. Because not all candidate fusions were tested by TaqMan assay, all fusion events from Tier-1, Tier-2 as well as TaqMan supported Tier-3 from the final candidate fusion list (Table S1) were used in stratification regardless of TaqMan results. In view of the limited number of fusions detected in the Rush dataset we evaluated just 2 categories: fusion detected or not detected, whereas in the Providence dataset we evaluated four abundance categories. The 8 patients with more than two fusions (subsequently referred to as multiple fusions) in Providence exhibited a statistically significant increased recurrence risk compared to patients from the three other groups having fewer detected fusion genes (Figure 3B). In the Rush dataset disease recurred at an increased rate among patients with detected fusions, although this relationship does not achieve statistical significance, possibly due to the limited fusion number detected from the low quality FFPE samples. Recognizing that including predicted fusion transcripts in this analysis necessarily reduces confidence in it, we also evaluated only the 36 TaqMan supported fusion transcripts from the Providence cohort. Figure 3C shows that a similar trend is still observed. The Rush data set yields only 11 TaqMan supported fusion transcripts which are too few to generate a meaningful Kaplan-Meier plot. To check whether minimizing the FFPE block age effect alters this relationship, we grouped patients into either upper or lower quartiles of the block age (binning patients with comparable block age or adjusting fusion numbers by RNA-Seq quality was not meaningful given the small numbers of patients and limited fusion numbers in these cohorts). The correction by sub-setting strengthens the association between fusion number and recurrence risk for both cohorts (Figure S2).

## Discussion

We present here novel evidence that increasing frequency of fusion transcripts is associated with poor prognosis. This study also adds to the molecular knowledge of breast cancer complexity by identifying 118 candidate fusion transcripts and many TaqMan supported fusion transcripts, all of which are novel except TFG->GPR128. Moreover, these fusions could be detected in single-end RNA-Seq data from aged FFPE tumor tissues by applying gFuse, a cohort based bioinformatics method. Among the total 118 candidate fusion transcripts identified, 3 unique fused gene pairs were recurrent and supported by TaqMan in the two cohorts of 212 total patients. The rate at which recurrent fusions were observed and the general novelty of the observed fusion transcripts in this study are in line with the previous publications about the very low recurrence of fusions in solid tumors such as 2–7% EML4-ALK in non-small lung cancer patients [4], [5]. It is notable that the recent TCGA (The Cancer Genome Atlas) consortium efforts with large patient cohorts and fresh frozen samples assisted with whole genome sequencing identified primarily private (found in one sample only) fusion transcripts [27], [31]–[33]. In 416 clear cell renal carcinoma patients, 70 out of 83 fusion transcripts are private [27]. In 322 endometrial carcinoma patients, 47 out of 49 fusion transcripts are non-recurrent [31]. In 97 colorectal cancer patients, 35 out of 38 fusion transcripts predicted from DNA translocations only exist in one patient [32].

An important feature of the fusion transcript detection pipeline described here is the use of expression profiling to nominate candidate fusion transcripts from RNA-Seq data that has sparse coverage of fusion junctions. With the dataset analyzed here, this step (Step 7) retains 8% of fusion candidates (Figure 1A). Generally, pathologically important gene fusions in cancer are characterized by one gene that is expressed at relatively high levels in non-fused state fused to another gene that is expressed at relatively low levels in non-fused state, the strong promoter of the 5′ gene up-regulates expression of an oncogenic 3′ gene (“oncogenic gene fusion model”) [34]. This predicts discontinuous expression patterns could be observed at either 5′ donor or 3′ acceptor fusion junction sites. Among 31 TaqMan supported high confidence Tier-1 fusions identified here (Figure 1B), 77% of them exhibit such interrupted expression patterns at fusion junctions (mostly acceptor junctions), consistent with the oncogenic gene fusion model. It is also possible that the gene expression filter removes a percentage of true fusion transcripts. When we performed TaqMan assays on a few fusion candidates that had single non-redundant reads without interrupted expression patterns, only one (ESR1->C6orf21) was supported by TaqMan. It is likely that in many cases fusion gene candidates removed by the gene expression filter that represent true fusion events are expressed at low levels. While it seems plausible that such fusion genes have little or no influence on tumor behavior, in fact their contribution is unknown.

To tailor this method to the short insert size and low complexity of FFPE RNA-Seq data, the candidate fusion templates are extended across a cohort or from one cohort to another to maximize the probability of identifying recurrent fusions. The potential of the cohort-based approach was demonstrated by our identification of a total of 6 recurrent TFG->GPR128 fusions across two cohorts, which include 1 Tier-1 fusion, 3 Tier-2 fusions, and 2 Tier-3 fusions (Table S1). The Tier-1 fusion was initially identified in a Rush sample, and extension of the Rush fusion templates to the Providence cohort allowed us to identify one Providence Tier-2 fusion, in which a single unique read split across the fusion junction with only 10 bp aligned to its acceptor gene. Sequence alignment tools cannot positively align a 10 bp sequence to its correct position in a whole genome, but this targeted exploration of candidate fusion sites allowed us to recognize recurrent events that were missed in the individual sample analysis. Further, assisted by the expression profiling analysis, another Tier-3 fusion was predicted in the Providence cohort.

Our method also addresses intronic RNA sequences, in recognition of the large amount of intronic sequence information present in FFPE RNA. Both donor and acceptor pre-mRNAs are built into 5 template sets to filter out reads mapped to mRNA precursors. On the other hand, the introns are selectively included in the expression profiling analysis to take advantage of abundant intronic sequence information. The two different remapping steps by GSNAP (Step 2 and Step 5 in Figure 1A) were designed to improve the mapping accuracy given the short inset size of FFPE. The success of these FFPE RNA-targeted designs is reflected by the high frequency of TaqMan support rates in the Tier-1 category (Figure 1B).

Although the cohort based strategy described here was developed with and applied to FFPE tissue and single end RNA-Seq datasets, it is also relevant to fusion transcript detection in cell lines and fresh frozen samples. Single molecule sequencing and other long read approaches aimed at increasing read length are expected to generally improve detection of genomic rearrangements, but the benefit of these improvements for FFPE specimens will be limited due to the short RNA fragments isolated from archived FFPE samples. Rapidly decreasing sequencing costs will enable data collection on more archived FFPE samples, therefore we anticipate that the method presented here will continue to facilitate fusion transcript detection and biomarker discovery in FFPE RNA.

Fusion transcripts may result from genuine genomic rearrangements or transcript level rearrangements such as trans-splicing [35]. One type of widely occurring, but biologically irrelevant trans-splicing, is a reverse transcriptase (RT) artifact derived from sequence homology [36]. Although our method doesn't distinguish genuine genomic rearrangement-derived gene fusions from trans-splicing derived fusions, there is no evidence of RT derived fusion artifacts in our study. First, our method searches for template sequence homologies to effectively remove false positive fusions generated by mapping algorithm or RT errors. Second, the identified fusions have canonical splicing tags while non-canonical splicing is characteristic of RT-derived trans-splicing [36]. Further evidence against RT based trans-splicing artifacts in this study comes from our TaqMan assay results. TaqMan assays were run against amplified RNA samples that shared the same source RNA as the RNA-Seq libraries but were prepared independently. Systematic RT errors would generate dis-concordance between the fusion calls made by the RNA-Seq fusion detection pipeline and TaqMan assays, but fusion transcripts identified by our pipeline and by the TaqMan assays are completely concordant (Table S2). Taken together, these data suggest that the fusion events we identified are unlikely to be due to artifactual trans-splicing events during RNA-Seq library preparation and thus represent bona-fide fusions of genomic or transcriptomic origin. We do acknowledge that the TaqMan assays can tolerate a few single nucleotide variants within the assayed amplicons and, while we think it is unlikely, it is conceivable that some of the identified fusion transcripts are not accurate.

Here we have observed a substantially higher percentage of intronic reads (∼60%) than what have been reported in many studies using fresh tissue RNA [37]. We believe this is explained by an intron sequence enrichment that occurs as a result of formalin fixation of RNA. We note that in another study using FFPE tissues more than 50% of the reads are intronic [38]. We have excluded the possibility of genomic DNA (gDNA) contamination in our FFPE RNA preparations by use of criteria: DNAase I treatment, and confirmation by TaqMan assays for gDNA (Table S4). The increased proportion of intronic reads from FFPE specimens may reflect selective degradation of cytoplasmic RNA (i.e., non-intronic RNA) by RNase during formalin fixation [39].

This study demonstrates the technical feasibility and potential biomedical value of being able to detect fusion transcripts in archival tumor specimens having attached clinical records. Although the average frequency of detected fusion transcripts is relatively low per patient, plausibly attributable to the low quality of FFPE RNA-Seq libraries, the frequency of fusion events found in our cohort nevertheless appears to have prognostic significance. Many of the identified fusion partner genes belong to the kinase, phosphatase and ubiquitin ligase families, which are attractive pharmaceutical targets in oncology. Both fusion frequency and tumor prognosis may be linked to cancer genome instability, which can generate chromosome rearrangements and fusion transcripts. In conclusion, this study significantly enriches the current understanding of breast tumor complexity by discovering a large number of novel fusion transcripts. It confirms one of the challenges of cancer therapeutics, namely that each cancer is different and personalized treatment is needed. In parallel we demonstrate a unique approach that reveals the genetic compositions of individual cancers employing short read sequencing methods and bioinformatics analysis adapted for FFPE tumor tissues.

## Supporting Information

Figure S1 Protein domains of fusion ESR1->AKAP12 are illustrated based on UniProt database (www.uniprot.org). The red vertical line indicates the fusion position on the corresponding protein. The amino acid length and amino acid positions of each fusion position are labeled on the top of each protein. A. The protein domains of ESR1 protein P03372 (UniProt ID). B. The protein domains of AKAP12 protein Q02952 (UniProt ID). C. The protein domains of two predicted fusion protein isoforms ESR1->AKAP12. The one amino acid insertion generated from the fusion event is labeled on each fusion protein.
(EPS)

Figure S2 Kaplan-Meier plots of patient subsets of Providence or Rush patients as a function of fusion numbers, segregated by block age. Either upper quartile or lower quartile based on block age is selected to examine the effect of the block ages on the disease outcome. The log-rank p-values are displayed.
(EPS)

Table S1 An excel file contains the complete information of all 118 fusion transcripts from Providence and Rush cohorts. All Tier-1 and Tier-2 fusions from Figure 2B are included regardless of TaqMan status. The splicing consensus sequences are included for47 TaqMan supported fusion transcripts, which all contain the splicing tag GU-AG.
(XLSX)

Table S2 An excel file contains all TaqMan results.
(XLSX)

Table S3 An excel file contains the complete information of 19 fusion junction sequences confirmed by PGM.
(XLSX)

Table S4 TaqMan assays for examination of residual gDNA contamination of all Providence and Rush cohorts using beta-actin. Each sample from Providence has 6 TaqMan replicates, and each sample from Rush has 3 TaqMan replicates. Each TaqMan plate has Human Genomic DNA (Promega Corporation, Madison, WI) triplicates as positive control, and no template triplicates as negative control. A second DNase I treatment was repeated on 2 Providence RNA samples which didn't pass the first residual gDNA contamination assays.
(XLSX)
